# Leptin Receptor (rs1137101) and Brain-Derived Neurotrophic Factor (rs925946) Gene Variants Are Associated with Obesity in the Early- but Not in the Late-Onset Population of Hungarian Psoriatic Patients

**DOI:** 10.3390/life11101086

**Published:** 2021-10-14

**Authors:** Zita Szentkereszty-Kovács, Szilvia Fiatal, Eszter Anna Janka, Dóra Kovács, Andrea Szegedi, Éva Remenyik, Dániel Törőcsik

**Affiliations:** 1 Department of Dermatology, Faculty of Medicine, University of Debrecen, Nagyerdei Krt. 98, 4032 Debrecen, Hungary; szentkeresztykovacs.zita@med.unideb.hu (Z.S.-K.); janka.eszter@med.unideb.hu (E.A.J.); kovacs.dora@med.unideb.hu (D.K.); aszegedi@med.unideb.hu (A.S.); remenyik@med.unideb.hu (É.R.); 2 Department of Public Health and Epidemiology, Faculty of Medicine, University of Debrecen, Kassai út 26, 4012 Debrecen, Hungary; fiatal.szilvia@med.unideb.hu

**Keywords:** psoriasis, obesity, leptin receptor, brain-derived neurotrophic factor

## Abstract

Background: Psoriatic patients have considerably higher odds of being obese compared with the general population; however, the exact pathophysiological link between psoriasis and obesity needs to be elucidated. Methods: To investigate the association of psoriasis with established obesity-related gene variants, we conducted a population-based case-control study including 3541 subjects (574 psoriasis cases and 2967 controls from the general Hungarian population). Genotyping of 20 SNPs at ADIPOQ, BDNF, FTO, GNPDA2, LEPR, MC4R, NEGR1, NPY, PPARG, TMEM18, and UCP2 were determined, and differences in genotype and allele distributions were investigated. Multiple logistic regression analyses were implemented. Results: Analysis revealed an association between the G allele of the rs1137101 polymorphism (LEPR gene) and obesity risk (OR: 3.30 (1.45; 7.50), *p* = 0.004) in the early-onset group of psoriatic patients. Furthermore, the T allele of rs925946 polymorphism (BDNF gene) was also associated with increased risk of obesity in early-onset psoriasis (OR: 2.26 (1.24; 4.14), *p* = 0.008). Conclusions: Our results suggest that in psoriatic patients, there are prominent differences in the causes of obesity that should be accounted for, including not only environmental factors but also patient characteristics, such as the time of disease onset as well as genetic factors.

## 1. Introduction

Psoriasis is a chronic, immune-mediated non-infectious systemic inflammatory disease with a prevalence of 1–4% in the general population, having genetic as well as environmental contributing factors [1]. Regarding its clinical phenotypes, chronic plaque-type, guttate, erythrodermic, and pustular psoriasis can be distinguished that may be present with various levels of severity. While based on the localization, other variants such as inverse or flexural psoriasis, sebopsoriasis, palmoplantar psoriasis, and nail psoriasis can be described as well [2]. According to the age of onset, early-onset (≤ 40 years) and late-onset (>40 years) of the disease can be subclassified, whereas early-onset also reflects the predomination of genetic factors in the development of the disease [3].

Besides it’s severe impact on the quality of life and the success of the targeted therapies that have resulted from the breakthrough in our understanding of psoriasis pathogenesis at cellular and molecular levels, the diversified manifestations of the associated comorbidities have also positioned psoriasis at the center of dermatology. Epidemiological studies revealed that while psoriatic arthritis is present in nearly 10% of the patients, metabolic syndrome (MetS), which includes the clustering of insulin resistance, dyslipidaemia, hypertension, and obesity, has a pooled odds ratio (OR) of 2.14 in psoriatic patients in comparison to the general population [4,5,6]. Moreover, the prevalence of MetS was significantly increased even among patients with psoriatic arthritis (PsA) when compared to the rheumatoid arthritis (RA) population, as revealed in a meta-analysis by Loganathan et al. [7]. Importantly, obesity is not only involved in sustaining a low-grade inflammation through the production of cytokines such as TNF-α and IL-6, which may contribute to the increased severity of psoriasis and PsA symptoms, but may also modify the effect of therapies, especially of TNF-α inhibitors [7,8]. Despite these findings, although patients are routinely screened and treated for psoriatic arthritis, for other common comorbidities such as cardiovascular disorders and MetS, they are still not properly screened for and managed [5], which may be partially explained by the fact that the question of whether MetS is indeed associated with psoriasis at the level of pathogenesis or is perhaps more a result of impaired life quality of patients leading to behavior changes is yet to be answered in full.

In our previous study, which investigated the clinical and epidemiological characteristics as well as the comorbidities in our psoriasis population at the University of Debrecen in Hungary, we found obesity to be more common in the late- compared to the early-onset psoriasis group, particularly in elderly patients [9]. Although our results suggested that the underlying common inflammatory pathways and environmental factors such as lifestyle may be pivotal in the obesity of psoriatic patients, some degree of common genetic determination could not be ruled out. Such a role for genetic factors was supported by systemic reviews and meta-analyses, as well as by the results of a cross-sectional, population-based twin study of 37,481 Danish twins including psoriasis-discordant (dizygotic) twin pairs, which found obesity more common in the twin with psoriasis compared with the one without psoriasis, while in monozygotic twin pairs, less correlation was found between obesity and psoriasis [10]. However, to define the exact role for genetic components in a disease that is mostly multifactorial, such as obesity, is a great challenge. Out of the 870 single nucleotide polymorphisms (SNPs) identified so far as being associated with obesity, only 5% showed a direct link with the increase in the BMI score [11]. Most of these SNPs are in genes related to the regulation of appetite and satiety at the CNS level (e.g., BDNF, LEPR, MC4R, NEGR1, NPY, TMEM18) [12,13,14,15,16], insulin secretion and action (e.g., ADIPOQ) [12,17], adipogenesis (e.g., PPARG) [18,19], and energy and lipid metabolism (e.g., FTO, UPC2) [12,20].

In this study, therefore, we aimed to further analyze our psoriasis cohort to address if genetic factors driving obesity could be linked to psoriasis by using samples from the same biobank that was used also to investigate the association of psoriasis with alcohol consumption and dependence-related gene variants [21]. Selecting 20 SNPs of candidate genes associated with obesity that were previously assessed in the general Hungarian population [22], we investigated and compared their distribution and association with obesity in 574 psoriatic patients and 2967 subjects from the general Hungarian population.

## 2. Materials and Methods

A total of 574 patients diagnosed with psoriasis vulgaris were enrolled in the study. The diagnosis was approved by at least two dermatologists. Data, including family history (familial aggregation was positive if psoriasis existed in at least one more case among first degree relatives extended to grandparents in familial anamnestic data) and age of onset (early-onset:  ≤ 40 years, late-onset  > 40 years), were collected. To assess the severity of psoriasis, a Psoriasis Area and Severity Index (PASI) score was used. Symptoms were dichotomized as follows: in the case of patients receiving only topical treatment and/or having a PASI  < 10 without therapy, the disease was specified as mild psoriasis; a score of PASI ≥  10 was specified as severe psoriasis. Patients receiving systemic therapy were all included in the severe psoriasis group independent of the recent PASI score. Body mass index (BMI) categories were divided into three categories (normal weight: BMI < 25, overweight: 25 ≤ BMI < 30, and obese: BMI ≥ 30).

Sample representative of the Hungarian General (HG) population in terms of geographic, age, and sex distributions were obtained from a population-based disease registry, the General Practitioners’ Morbidity Sentinel Stations Program (GPMSSP) [23]. Details of sampling methodology and data collection are described elsewhere [24].

Informed consent was obtained from all individual participants included in the study. The study protocol was approved by the Regional Institutional Scientific and Research Ethical Board of the University of Debrecen, Hungary in accordance with the principles from the Declaration of Helsinki (44753/2012/EKU; 21 November 2012).

### 2.1. SNP Selection

Our study utilized the same collection set of 20 SNPs in candidate genes likely to be associated with the development of obesity that was previously assessed in the HG population [22]. SNP selection, with an emphasis on GWAS (PubMed) data, was based on a systematic literature review that revealed which SNPs showed significant associations with obesity-related features and had a minor-allele frequency >5% in the HapMap dataset for a European ancestry population sample (CEU) (www.hapmap.org, accessed on 3 December 2016).

### 2.2. DNA Preparation

DNA isolation was performed from ethylenediaminetetraacetic acid-anticoagulated blood samples using the MagNA Pure LC DNA Isolation Kit—Large Volume (Roche Diagnostics, Mannheim, Germany) according to the manufacturer’s protocol. The extracted DNA samples were eluted in a MagNA Pure LC DNA Isolation Kit-Large Volume Elution Buffer (Roche Diagnostics, Basel, Switzerland) and stored at −30 °C until measurements were carried out.

### 2.3. Genotype Assessment

Genotyping was performed by the Mutation Analysis Core Facility (MAF) of the Karolinska University Hospital (Stockholm, Sweden) using the Sequenom Mass ARRAY platform (Sequenom Inc., San Diego, CA, USA) with iPLEX Gold chemistry. Validation, concordance analysis, and quality control were conducted by the MAF according to their protocols, resulting in a successful genotyping outcome for 574 psoriatic and 2967 HG DNA samples.

### 2.4. Statistical Analyses

The data were analyzed using STATA 12.0 Statistical software (StataCorp LP, College Station, TX, USA) and by SPSS 25 (SPSS package for Windows, Release 25; SPSS Inc., Chicago, IL, USA). The Mann–Whitney U and χ^2^ tests were used to compare the mean age distribution of the two study groups. The existence of the Hardy–Weinberg equilibrium (HWE) and significant differences in the allele and genotype frequencies between the two populations were examined with the χ^2^ test. To decrease the proportion of false positive results, a p threshold of 0.002 was applied (Bonferroni correction); otherwise, the threshold for significance was 0.05.

To take account of confounding effects of gender and age on differences between study populations, linear regression models were constructed. Psoriatic samples were divided into several subgroups defined by clinical parameters such as familial aggregation, age at onset, and severity as described previously. Association analyses were performed according to an additive model using age and sex as covariates by using PLINK v1.07 software (Center of Human Genetic Research (CHGR), Boston, MA, USA). For the power calculation of the association analyses, the Quanto version 1.2 software (Department of Population and Public Health Sciences, Keck School of Medicine of University Southern California, Los Angeles, CA, USA) was used.

To determine the association of genotype, onset of psoriasis, severity, age, and gender with BMI categories (normal weight, overweight, obese), multinomial logistic regression analyses (odds ratio (OR) with corresponding 95% upper and lower confidence intervals (95% CI)) was performed. The significance level was set at 0.05.

## 3. Results

### 3.1. Characteristics of Study Populations

When compared to the HG population, the proportion of male individuals in the psoriatic group was significantly higher (psoriatic: 61.8% vs. HG: 46.8%, *p* < 0.001), while the mean age was 50.28 years ± 15.55 in the case of patients with psoriasis and 45.53 years ± 14.62 in the case of the HG population. The BMI distribution was not different in the study populations (psoriatic: 30.0 kg/m^2^ (SD ± 6.5) vs. HG: 29.3 kg/m^2^ (SD ± 6.9)). 

Stratifying psoriatic patients to early-onset (≤40 years) and late-onset (>40 years) groups, the mean age among the early-onset was significantly lower than in the late-onset group according to the Mann–Whitney U test (*p* < 0.001). Comparing familial aggregation and sporadic occurrence between early- and late-onset groups showed that the sporadic occurrence was significantly higher among the late-onset group, while the familial aggregation showed significantly greater incidence in the early-onset group using chi^2^ statistics (*p* < 0.001). With regard to BMI, in accordance with our previous study [9], the proportions of obese patients were significantly higher in the late-onset psoriasis group. In respect of gender and severity, there were no differences between the two subgroups (Supplementary Table S1).

### 3.2. Frequency and Impact of the Selected SNPs in the Study Populations

All analyzed obesity-predisposing SNPs were in Hardy–Weinberg equilibrium in the two study groups of HG and psoriasis. Risk allele frequencies showed no significant differences between the two study groups, even when further subgroups of psoriatic patients were created (early- vs. late-onset psoriasis) (Supplementary Table S2).

Although the sample size showed rather low power for all the SNPs (6–46%), suggesting that they have a weak contribution to the development of obesity, statistically significant associations were still found in the psoriasis populations. Both the LEPR gene variant (rs1137101) and the BDNF gene variant (rs925946) showed strong association with obesity in the association analysis, as indicated by the beta values (1.068 (0.360; 1.777I), *p* = 0.003; and 1.237 (0.414; 2.059), *p* = 0.003, respectively). 

A further association signal was found for SNPs in the FTO gene (rs1558902, ß = 0.407, 95%CI (0.137; 0.677), *p* = 0.0032; rs1121980, ß = 0.446 (0.176; 0.715), *p* = 0.0012; rs9939609, ß= 0.410, 95% (0.139; 0.681); *p* = 0.003; rs9941349, ß = 0.434 95% (0.163; 0.706), *p* = 0.0017) and for the MC4R gene (rs17782313; ß = 0.457 95% CI (0.136; 0.779), *p* = 0.0053; rs12970134, ß = 0.463 95% CI (0.150; 0.775), *p* = 0.0037) in the general population, which is in contrast to previous studies that found an association of rs9939609 (FTO) and rs17782313 (MC4R) with obesity and psoriasis, although in a genetically different cohort of Polish [25] and Romanian [26] psoriatic patients. However, due to the low statistical power, we did not perform further analyses on them (Table 1).

### 3.3. LEPR rs1137101 Is Associated with Obesity in the Early- but Not in the Late-Onset Psoriasis Group

LEPR rs1137101 was further analyzed in psoriasis sub-populations based on BMI values, such as normal weight (reference), overweight, and obese. In the case of the GG genotype, a significant association with obesity was found when comparing the obese population to the normal weight subgroup (OR: 2.67 (1.34; 5.31), *p* = 0.005) (Table 2).

Next, we aimed to assess if the association of rs1137101 with obesity also showed a correlation with the onset of psoriasis; therefore, we stratified and compared the early-onset (≤40 years) and late-onset (>40 years) psoriasis groups. We found that the GG genotype distribution remained significant in the early- but not in the late-onset obese subgroup. Interestingly, just as in the whole psoriasis population group, the association between rs1137101 and obesity was independent of gender or disease severity (Table 3).

**Figure 1:**
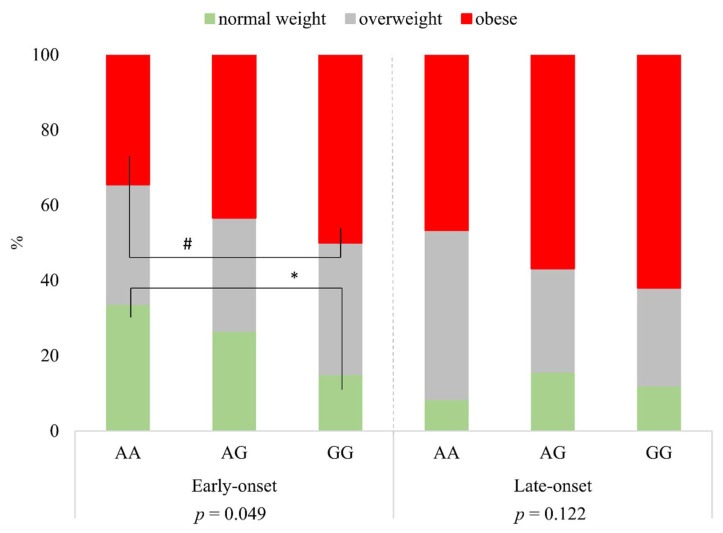
The distribution of BMI categories according to rs1137101 genotypes in early- and late-onset psoriatic patients. * significant; # borderline significant.

Investigating the distribution of BMI categories in respect of rs1137101 genotypes AA, AG, and GG, there were significant differences within the group of early-onset psoriatic patients (*p* = 0.049). While the proportion of patients with normal weight was significantly less in subjects with two risk alleles (GG genotype) than in the group of patients with two wild alleles (AA genotype (*)), the proportion of obesity was higher in the case of the GG genotype compared to the AA genotype (based on 95% confidence intervals, the difference was borderline significance (#)). In the case of the group of late-onset psoriatic patients, there were no significant differences between BMI categories and the genotypes (*p* = 0.122) (Figure 1).

It was also observed in our cohort that a familial predisposition for psoriasis is more frequent in the early- than in the late-onset subgroup (37.5% vs. 16.7%); therefore, we aimed to exclude the possibility of a familial accumulation for the GG genotype. In our analyses, no differences were found between the frequency of genotypes when subgroups of patients with sporadic and familial history were compared either in the early- or in the late-onset psoriatic groups (Supplementary Figure S1).

### 3.4. BDNF rs925946 Is Associated with Obesity in the Early- but Not in the Late-Onset Psoriasis Group 

In the case of rs925946 in the BDNF gene, the TG genotype (risk allele is T) showed significant association with obesity among psoriatic patients. A significantly higher prevalence of the TG subjects was found in the obese and in the overweight populations compared to the normal weight population (OR: 2.02 (1.21; 3.35), *p* = 0.007, OR: 1.91 (1.14; 3.19), *p* = 0.013) (Table 4).

Assessing the differences between the early- and the late-onset subgroups, the occurrence of the TG genotype (but not the TT) was significantly higher in the early-onset subgroup both in the overweight and obese categories compared to normal weight (OR: 2.08 (1.12; 3.84), *p* = 0.020; OR:2.26 (1.24; 4.14), *p* = 0.008). The association between rs925946 and obesity was also independent of gender or disease severity (Table 5).

**Figure 2:**
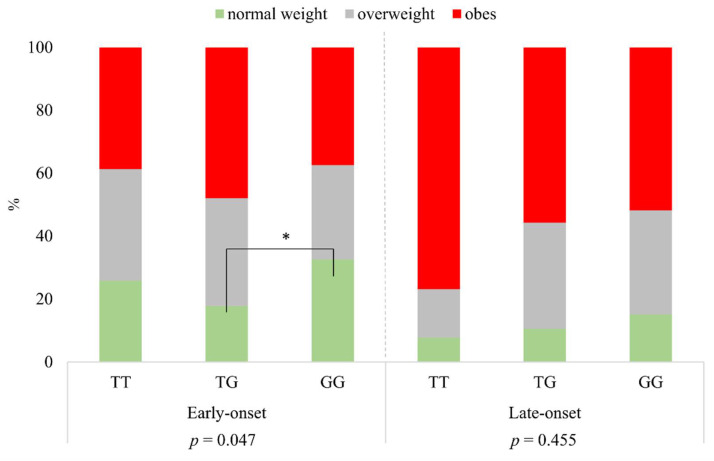
The distribution of BMI categories according to rs925946 genotypes in early- and late-onset psoriatic patients. * significant.

Defining the BMI categories according to rs925946 genotypes of TT, TG, and GG, a significant difference was found in the group of early-onset psoriatic patients (*p* = 0.047). The proportion of normal weight was significantly higher in the case of GG than in that of subjects with the TG genotype. In the group of late-onset psoriatic patients, there was no significant difference between the different BMI categories and genotypes (*p* = 0.455) (Figure 2).

## 4. Discussion

The results of our study suggest that although obesity is more frequent in the late-onset group of psoriatic patients, for an obese patient belonging to the early-onset group, there is a higher chance to have risk alterations in the LEPR rs1137101 and in the BDNF rs925946 polymorphisms compared to an obese patient from the late-onset or from the general Hungarian population. Based on our findings, in the case of patients with psoriasis, there may be prominent differences in the background of obesity, which could influence not only further patient characteristics but could also have an impact on the manifestation of psoriasis. However, the limitations of our study should be kept in mind, as the group comes from a single clinic and from a single geographic area of Hungary. 

Based on the principal biological properties of the various bioactive proteins secreted by adipocytes—the adipokines [27]—with respect to the regulation of immune responses locally and systemically, adipokines play a key role in the so-called immune-metabolic dialog [28]. Although the family of adipokines includes members with pro- as well as anti-inflammatory properties, the increased amount of the white adipose tissue (WAT) altogether leads to a strengthened pro-inflammatory state, which is pathognomonic in the manifestation of psoriasis [29].

Leptin, an inflammatory adipokine, has a primary role to regulate weight by acting on its receptors in the hypothalamus; however, it is also known to link lipid metabolism with inflammation [30]. Of the LEPR, multiple splice variants have been identified, but only the long isoform (Ob-Rb) can induce pathways that result in the activation of the nuclear factor kappa-B (NF-κB), which is the major transcription regulator of inflammatory mediators that are also active in psoriasis. Such pathways include the Janus kinase 2/signal transducer, the activator of transcription factor 3 (JAK2/STAT3) signaling [31,32], and the mitogen-activated protein kinase (MAPK) family (p38 MAPK), as well as the stress activated c-Jun N-terminal kinase (JNK) and phosphatidylinositol 3-kinase/protein kinase B (PI3K/Akt) pathways [31,33], which together explain the multiplex inflammatory responses and the wide repertoire of the responding cells in the skin that are not only immune cells but also keratinocytes, fibroblasts, and sebocytes [34]. Importantly, besides murine studies describing an increased inflammation in the imiquimod-induced psoriasis-like skin in leptin deficient (ob/ob) mice [35,36], LEPR together with leptin was found to have significantly higher expression levels in the skin of severe psoriatic patients, even with normal BMI, compared to patients with mild–moderate psoriasis and controls in histological studies [28]. Moreover, leptin mRNA expression in the subcutaneous adipose tissue also positively correlated with the severity of psoriasis and the BMI in obese psoriatic patients [37] suggesting that leptin-induced signaling mediates both local as well as systemic changes. Still, at least to our knowledge, no polymorphisms in the gene encoding LEPR has been linked to psoriasis so far, while in the case of the gene encoding leptin, rs7799039 was associated with the plasma leptin levels and the metabolic syndrome with psoriasis [38]. However, the association of rs2060713 polymorphism and psoriasis vulgaris showed a trend in males with only the early-onset type of psoriasis and in females with the late-onset type of the disease [39]. Such contradictory results are also common in other studies, such as those assessing serum adipokine levels. While some studies found elevated leptin serum levels even in psoriatic patients with normal BMI, showing a correlation with PASI scores [28,40,41], studies are also available with opposing data [42,43]. The contradiction may arise from the selected polymorphisms, the various study populations and from molecular interactions that could influence leptin levels [44], as well as from the different stratifications that are very often challenging due to the complexity of psoriasis symptoms and the heterogeneity of patients. In our recent study, therefore, we applied a previously verified panel of genetic variants identified from genetic association studies focused primarily on European populations [45]; we also extended our study of a detailed psoriasis cohort that has been used for our previous publications [9,21] and included data about the obesity related parameters for all patients. 

Our findings, that the most frequently investigated SNP among LEPR polymorphisms, rs1137101, was associated with obesity in the early-onset group of psoriatic patients, complement previous studies which examined the relationship between psoriasis and the plasma or the tissue expression levels of different adipokines [9,29]. Moreover, our study provided further evidence that adipokines, particularly leptin, could have an important role in disease pathogenesis and be associated with systemic comorbidities, such as obesity, which may be determined already at the level of the genetic background. Therefore, the involved patients in the early-onset psoriasis group should be approached differently as regards lifestyle recommendations. Further findings in certain populations—that besides obesity, rs1137101 has been linked to type 2 diabetes mellitus [46], various types of cancer such as endometrial [47], prostate [48], colorectal [49] and renal cell carcinoma [50] as well as to keloid formation [51], multiple sclerosis severity score [52], the outcome of renal transplantation [53] and to susceptibility to polycystic ovary syndrome [54]—suggests that sub-stratification and follow up of the involved individuals may also be a promising basis for prevention strategies.

Neurotrophins (e.g., brain-derived neurotrophic factor (BDNF]) play an important role in regulating energy and glucose homeostasis (for example, in cholesterol metabolism) not only in neuronal differentiation [55] but also in peripheral tissues [56]; therefore, they are also associated with inflammatory diseases, as well as MetS [57,58,59,60]. BDNF activity is mediated through the TrkB receptor [61] and the neurotrophin receptor p75 (p75NTR), also known as the low-affinity nerve growth factor receptor, which belongs to the TNF-receptor superfamily [62]. Regarding psoriasis, BDNF was found to be crucial in the maintenance of normal keratinocyte apoptosis and epidermal homeostasis [63], while p75NTR protein was found to be absent in lesional psoriasis skin. Regarding its serum levels as well as its staining intensities, significantly lower levels were detected in both the epidermis and the dermis of psoriatic patients compared to the control group; however, no difference was observed between the psoriasis patients with and without MetS [63,64,65]. Interestingly, in assessing the polymorphism rs6265 in the BDNF gene, its combined effect with higher BMI was found to increase the risk and clinical severity of psoriasis in the Chinese Han population [66]; nevertheless, as shown in both this and our previous [21] studies, we found no significant results in the case of rs6265 in our psoriasis cohort. Our findings that rs925946 showed a strong association with obesity, however, supports the theory that BDNF may indeed be involved in linking obesity with psoriasis in the early-onset group of patients. Further findings—that the blood levels of BDNF were found to be reduced not only in psoriasis but also in depression, and thus may link major depression with psoriasis—call for studies to also integrate BDNF into psycho-dermatology [67,68,69]. However, the stigma of depression, which is similar to what we experience in regard to alcohol consumption [70], greatly limits the data collection for such studies. Still, it is tempting to challenge BDNF as a possible target that could modify obesity, psoriasis severity, and the related depression at once [71]. 

Based on our results, we suggest that genetic predisposition should be taken into account not only when addressing psoriasis, but also when the related obesity too. However, to translate this knowledge into understanding the association of obesity with psoriasis, such as to develop a holistic treatment strategy for a successful patient care and management with lifestyle interventions [72], consideration of individual patient characteristics is still pivotal. 

## Figures and Tables

**Table 1:** The effect alleles and the association measures (Beta) of selected obesity associated SNPs in the study groups.

Gene/SNP	Effect Allele	Hungarian General Population (*n* = 2967)			Psoriasis Group (*n* = 574)		
		Beta ^#^ (95% CI)	*p*-Value	Power *	Beta ^#^ (95% CI)	*p*-Value	Power *
*LEPR*							
**rs1137101**	G	0.031 (−0.242; 0.304)	0.8237	0.05	**1.068 (0.360; 1.777I)**	**0.003**	0.44
*NEGR1*							
rs2815752	A	0.187 (−0.102;0.477)	0.2049	0.09	0.504 (−0.265; 1.274I)	0.199	0.12
*TMEM18*							
rs2867125	C	0.269 (−0.079; 0.615)	0.13	0.12	−0.615 (−1.653; 0.422)	0.246	0.12
rs6548238	C	0.320 (−0.022; 0.663)	0.067	0.15	−0.397 (−1.441; 0.646)	0.456	0.08
*PPARG*							
rs1801282	C	−0.084 (−0.495; 0.327)	0.689	0.05	−0.539 (−1.641; 0.562)	0.337	0.09
*ADIPOQ*							
rs2241766	G	−0.114 (−0.560; 0.331)	0.6145	0.05	−1.159 (−2.449; 0.130)	0.078	0.20
rs1501299	T	−0.093 (−0.391; 0.204)	0.538	0.06	0.294 (−0.542; 1.129)	0.491	0.07
*GNPDA2*							
rs10938397	G	0. 248 (−0.029; 0.526)	0.079	0.15	0.039 (−0.692; 0.771)	0.916	0.05
*NPY*							
rs16139	C	0. 301 (−0.374; 0.976)	0.382	0.07	−1.362 (−3.057; 0.333)	0.116	0.17
*BDNF*							
**rs925946**	T	0.247 (−0.064; 0.559)	0.121	0.12	**1.237 (0.414; 2.059)**	**0.003**	0.46
rs6265	C	0.28 (−0.063; 0.623)	0.109	0.13	0.837 (−0.109; 1.783)	0.083	0.19
*UCP2*							
rs660339	G	−0.104 (0.386; 0.177)	0.468	0.07	−0.207 (−0.927; 0.513)	0.574	0.06
rs659366	C	−0.053 (−0.340; 0.234)	0.716	0.05	−0.319 (−1. 062; 0.424)	0.4	0.08
*FTO*							
rs6499640	A	0.172 (−0.102; 0.446)	0.219	0.09	0.825 (0.044; 1.06)	0.039	0.27
rs1558902	A	0.407 (0.137;0.677)	0.0032	0.31	0.323 (−0.454; 1.1)	0.415	0.08
rs1121980	A	0.446 (0.176; 0.715)	0.0012	0.36	0.460 (−0.316; 1.236)	0.246	0.12
rs9939609	A	0.410 (0.139; 0.681	0.003	0.31	0.358 (−0.413; 1.13)	0.363	0.09
rs9941349	T	0.434 (0.163; 0.706)	0.0017	0.34	0.522 (−0.249; 1.294)	0.185	0.14
*MC4R*							
rs17782313	C	0.457 (0.136; 0.779)	0.0053	0.28	0.418 (−0.452; 1.29)	0.346	0.09
rs12970134	A	0.463 (0.150; 0.775)	0.0037	0.3	0.232 (−0.632; 1.096)	0.599	0.06

^#^ Regression models were adjusted for age and sex and were based on a model assuming additive allelic effect. * For power calculation, the Quanto version 1.2 software was used. Bolds refer to the SNPs in case of significant results were found in the Psoriasis Group.

**Table 2:** The association between BMI categories and different variables among psoriatic patients in the case of rs1137101.

All Patients (*n* = 574)			
Reference: Normal Weight	Variables	OR (95% CI)	*p*-Value
overweight	age	1.02 (0.99; 1.04)	0.086
	gender (female/male)	0.66 (0.41; 1.08)	0.098
	genotype GG/AA	1.56 (0.78; 3.12)	0.208
	genotype AG/AA	0.89 (0.52; 1.51)	0.653
	onset (early/late)	0.75 (0.40; 1.41)	0.371
	severity (yes/no)	0.14 (0.64; 2.02)	0.649
obese	age	1.01 (0.99; 1.03)	0.629
	gender (female/male)	0.61 (0.38; 1.02)	0.056
	genotype GG/AA	**2.67 (1.34; 5.31)**	**0.005**
	genotype AG/AA	1.41 (0.82; 2.42)	0.211
	onset (early/late)	**0.59 (0.31; 0.98)**	**0.044**
	severity (yes/no)	1.19 (0.67; 2.10)	0.554

OR—odds ratio; CI—confidence intervals. Bolds refer to the variables in case of significant results were found in the Psoriasis Group.

**Table 3:** The association between BMI categories and different variables in early- and in late-onset psoriatic patients in the case of rs1137101.

		Early-Onset (*n* = 362)		Late-Onset (*n* = 212)	
Reference: Normal Weight	Variables	OR (95% CI)	*p*-Value	OR (95% CI)	*p*-Value
overweight	age	1.02 (1.00; 1.05)	0.057	1.03 (0.98; 1.08)	0.311
	gender (female/male)	0.61 (0.34; 1.09)	0.098	0.74 (0.29; 1.92)	0.538
	genotype GG/AA	**2.49 (1.09; 5.69)**	**0.031**	0.45 (0.11; 1.84)	0.265
	genotype AG/AA	1.21 (0.65; 2.28)	0.540	0.37 (0.11; 1.22)	0.103
	severity (yes/no)	1.23 (0.61; 2.48)	0.558	1.01 (0.34; 3.01)	0.991
obese	age	1.01 (0.98; 1.03)	0.494	1.01 (0.97; 1.06)	0.573
	gender (female/male)	0.60 (0.34; 1.07)	0.086	0.63 (0.25; 1.61)	0.336
	genotype GG/AA	**3.30 (1.45; 7.50)**	**0.004**	1.05 (0.26; 4.31)	0.947
	genotype AG/AA	1.57 (0.84; 2.95)	0.158	0.85 (0.26; 2.80)	0.784
	severity (yes/no)	1.05 (0.53; 2.09)	0.879	0.73 (0.24; 2.18)	0.568

OR—odds ratio; CI—confidence intervals. Bolds refer to the variables in case of significant results were found in the early-onset psoriasis Group.

**Table 4:** The association between BMI categories and different variables among psoriatic patients in the case of rs925946.

All Patients (*n* = 574)			
Reference: Normal Weight	Variables	OR (95% CI)	*p*-Value
overweight	age	1.02 (0.99; 1.04)	0.073
	gender (female/male)	0.67 (0.41; 1.09)	0.110
	genotype TT/GG	1.39 (0.55; 3.56)	0.487
	genotype TG/GG	**1.91 (1.14; 3.19)**	**0.013**
	onset (early/late)	0.77 (0.41; 1.45)	0.415
	severity (yes/no)	1.11 (0.62; 1.97)	0.725
obese	age	1.00 (0.98; 1.02)	0.727
	gender (female/male)	0.65 (0.40; 1.06)	0.082
	genotype TT/GG	1.65 (0.66; 4.08)	0.283
	genotype TG/GG	**2.02 (1.21; 3.35)**	**0.007**
	onset (early/late)	0.57 (0.31; 1.07)	0.082
	severity (yes/no)	1.26 (0.71; 2.25)	0.427

OR—odds ratio; CI—confidence intervals. Bolds are necessary, they refer to variables found significant among psoriatic patients in case of rs925946.

**Table 5:** The association between BMI categories and different variables in early- and late-onset psoriatic patients in the case of rs925946.

		Early-Onset (*n* = 362)		Late-Onset (*n* = 212)	
Reference: Normal Weight	Variables	OR (95% CI)	*p*-Value	OR (95% CI)	*p*-Value
overweight	age	1.02 (0.99; 1.04)	0.060	1.03 (0.98; 1.08)	0.258
	gender (female/male)	0.66 (0.36; 1.18)	0.158	0.69 (0.26; 1.78)	0.437
	genotype TT/GG	1.59 (0.58; 4.37)	0.366	0.97 (0.07; 14.13)	0.985
	genotype TG/GG	**2.08 (1.12; 3.84)**	**0.020**	1.53 (0.58; 4.04)	0.391
	severity (yes/no)	1.26 (0.62; 2.56)	0.523	0.99 (0.33; 2.93)	0.979
obese	age	1.00 (0.98; 1.03)	0.696	1.02 (0.97; 1.07)	0.545
	gender (female/male)	0.69 (0.39; 1.22)	0.202	0.64 (0.25; 1.63)	0.347
	genotype TT/GG	1.35 (0.48; 3.77)	0.571	2.81 (0.24; 33.16)	0.412
	genotype TG/GG	**2.26 (1.24; 4.14)**	**0.008**	1.52 (0.58; 3.98)	0.398
	severity (yes/no)	1.18 (0.59; 2.36)	0.644	1.50 (0.50; 4.54)	0.470

OR—odds ratio; CI—confidence intervals. Bolds are necessary, they refer to variables found significant among early-onset psoriatic patients in case of rs925946.

## Data Availability

The data presented in this study are available on request from the corresponding author.

## Supplementary Materials

The following are available online at https://www.mdpi.com/article/10.3390/life11101086/s1, Table S1: Patient characteristics; Table S2: The genes, the effect alleles and the comparison of allele frequencies in the study groups; Figure S1: The frequency of different genotypes in sporadic and familial form among early- and late-onset psoriatic patients.
