# Supplementary material for: Leptin Receptor (rs1137101) and Brain-Derived Neurotrophic Factor (rs925946) Gene Variants Are Associated with Obesity in the Early- but Not in the Late-Onset Population of Hungarian Psoriatic Patients
Source: Life (Basel). 2021 Oct 14;11(10):1086. doi: 10.3390/life11101086 (PMC8538564; doi:10.3390/life11101086)
Supplement: Supplementary file 1 [file life-11-01086-s001.zip › life-1409185-supplementary.pdf]

Supplementary Materilas: Leptin Receptor (rs1137101) and Brain-Derived Neurotrophic Factor (rs925946) Gene Variants Are Associated with Obesity in the Early- but Not in the Late-Onset Population of Hungarian Psoriatic Patients

Zita Szentkereszty-Kovács, Szilvia Fiatal, Eszter Anna Janka, Dóra Kovács, Andrea Szegedi, Éva Remenyik and Dániel Törőcsik

Table S1. Patient characteristics.

	All Patients	Early Onset	Late Onset	Early vs. Late Onset
	N = 574	*N* = 362	*N* = 212	*p*-value
Mean age (year) ± SD	50.29 ± 15.55	43.92 ± 14.95	61.18 ± 9.24	**<0.001**
Gender (%)				0.072
Male	355 (61.8%)	234 (64.6%)	121 (57.1%)	
Female	219 (38.2%)	128 (35.4%)	91 (42.9%)	
BMI (%)				**<0.001**
Normal weight	122 (21.2%)	95 (26.2%)	27 (12.7%)	
Overweight	183 (31.9%)	115 (31.8%)	68 (32.1%)	
Obese	269 (46.9%)	152 (42.0%)	117 (55.2%)	
Familial form (%)				**<0.001**
Sporadic	403 (70.2%)	226 (62.4%)	177 (83.5%)	
Familial	171 (29.8%)	136 (37.6%)	35 (16.5%)	
Severity (%)				0.550
Mild/Moderate	117 (20.4%)	71 (19.6%)	46 (21.7%)	
Severe	457 (79.6%)	291 (80.4%)	166 (78.3%)	

SD – standard deviation.

Table S2. The studied genes, the effect alleles and the comparison of allele frequencies in the study groups.

Gene/SNP	*Effect Allele*	*Hungarian General population (N = 2967)*	*Psoriatic Population (N = 574)*	*P-Value*
		Effect Allele Frequency	*Effect Allele Frequency*	
LEPR				
rs1137101	G	0.4584	0.4649	0.6667
NEGR1				
rs2815752	A	0.66	0.6906	0.03328
TMEM18				
rs2867125	C	0.8145	0.8456	0.007857
rs6548238	C	0.8137	0.8427	0.01307
PPARG				
rs1801282	C	0.8746	0.8661	0.4041
ADIPOQ				
rs2241766	G	0.1086	0.08843	0.02966
rs1501299	T	0.288	0.2785	0.4912
GNPDA2				
rs10938397	G	0.4539	0.4464	0.6204
NPY				
rs16139	C	0.04545	0.05013	0.4699

BDNF				
rs925946	T	0.2552	0.2711	0.2372
rs6265	C	0.8036	0.8195	0.1852
UCP2				
rs660339	G	0.5926	0.6053	0.3958
rs659366	C	0.632	0.6414	0.5323
FTO				
rs6499640	A	0.5878	0.6056	0.235
rs1558902	A	0.453	0.4544	0.9285
rs1121980	A	0.4682	0.4733	0.7343
rs9939609	A	0.438	0.4471	0.5528
rs9941349	T	0.447	0.453	0.22
MC4R				
rs17782313	C	0.222	0.2302	0.5219
rs12970134	A	0.2429	0.2464	0.792

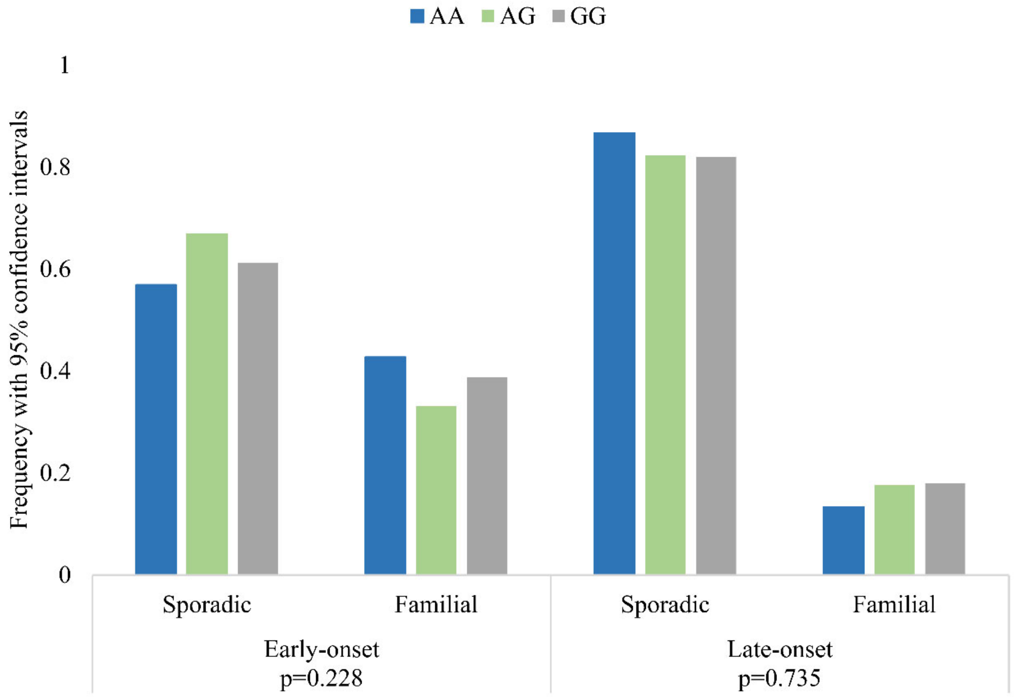

Figure S1. The frequency of different genotypes in sporadic and familial form among early- and late-onset psoriatic patients.
